# Oral Delivery of Nisin in Resistant Starch Based Matrices Alters the Gut Microbiota in Mice

**DOI:** 10.3389/fmicb.2018.01186

**Published:** 2018-06-15

**Authors:** Ronan Gough, Raúl Cabrera Rubio, Paula M. O'Connor, Fiona Crispie, André Brodkorb, Song Miao, Colin Hill, Reynolds P. Ross, Paul D. Cotter, Kanishka N. Nilaweera, Mary C. Rea

**Affiliations:** ^1^Teagasc Food Research Centre, Fermoy, Ireland; ^2^School of Microbiology, University College Cork, Cork, Ireland; ^3^APC Microbiome Ireland, University College Cork, Cork, Ireland; ^4^College of Science, Engineering, and Food Science, University College Cork, Cork, Ireland

**Keywords:** mouse, nisin, starch, resistant starch, microbiota, digestion, *Akkermansia*

## Abstract

There is a growing recognition of the role the gastrointestinal microbiota plays in health and disease. Ingested antimicrobial proteins and peptides have the potential to alter the gastrointestinal microbiota; particularly if protected from digestion. Nisin is an antimicrobial peptide that is used as a food preservative. This study examined the ability of nisin to affect the murine microbiota when fed to mice in two different starch based matrices; a starch dough comprising raw starch granules and a starch gel comprising starch that was gelatinized and retrograded. The effects of the two starch matrices by themselves on the microbiota were also examined. Following 16S rRNA compositional sequencing, beta diversity analysis highlighted a significant difference (*p* = 0.001, *n* = 10) in the murine microbiota between the four diet groups. The differences between the two nisin containing diets were mainly attributable to differences in the nisin release from the starch matrices while the differences between the carriers were mainly attributable to the type of resistant starch they possessed. Indeed, the differences in the relative abundance of several genera in the mice consuming the starch dough and starch gel diets, in particular *Akkermansia*, the relative abundance of which was 0.5 and 11.9%, respectively (*p* = 0.0002, *n* = 10), points to the potential value of resistance starch as a modulator of beneficial gut microbes. Intact nisin and nisin digestion products (in particular nisin fragment 22–31) were detected in the feces and the nisin was biologically active. However, despite a three-fold greater consumption of nisin in the group fed the nisin in starch dough diet, twice as much nisin was detected in the feces of the group which consumed the nisin in starch gel diet. In addition, the relative abundance of three times as many genera from the lower gastrointestinal tract (GIT) were significantly different (*p* < 0.001, *n* = 10) to the control for the group fed the nisin in starch gel diet, implying that the starch gel afforded a degree of protection from digestion to the nisin entrapped within it.

## Introduction

The gastrointestinal microbiota impacts on the health of the host in variety of ways, including through its potential to protect against infection, provide nutrients, and influence on bodyweight (Clarke et al., 2012; Nicholson et al., 2012; Jandhyala et al., 2015). The composition of the microbiota, and thus its health effects, can be altered by a variety of means, including antimicrobials and diet (Martínez et al., 2010; Cotter et al., 2012; Chung et al., 2016).

Nisin is an antimicrobial peptide with broad activity against Gram positive bacteria produced by strains of *Lactococcus lactis* subsp. *lactis* (Abee and Delves-Broughton, 2003). Nisin has been approved for use as a food preservative by both US Food and Drug Administration, (FDA) (US Food and Drug Administration, 1988) and by the European Food Safety Authority (EFSA) with its assigned E number being E 234 (Younes et al., 2017).

Nisin is very stable at low pH and at pH 3 there is <5% loss of activity when heated to 115°C for 20 min (Davies et al., 1998). However, while relatively resistant to passage through the acidic conditions in the stomach, nisin can be digested by pancreatin in the small intestine (Heinemann and Williams, 1966; Gough et al., 2017b), primarily by its trypsin and chymotrypsin components and therefore may not reach the lower gastrointestinal tract (GIT) in an intact form (Jarvis and Mahoney, 1969; Chan et al., 1996).

Few *in vivo* studies (Table 1) have investigated how dietary supplementation with nisin affects the microbiota of the lower GIT (Bernbom et al., 2006; Józefiak et al., 2013; Lauková et al., 2014; Kieronczyk et al., 2016) and no previous *in vivo* study has employed a high throughput sequencing (HTS)-based approach to examine the impact of nisin on the entire microbiota. Nisin has been consumed *in vivo* at up to 239 mg per kg body weight per day without any adverse effects on food consumption, body weight, hematology, ophthalmology, or gross pathology (Hagiwara et al., 2010). Although nisin doses of up to 173.9 mg per kg body weigh per day had no impact on the microbiota in a study on rats (Bernbom et al., 2006), nisin has been seen to influence the microbiota in some way in the majority of *in vivo* studies including those on mice, chickens and rabbits and in *in vitro* bovine and human microbiota studies (Table 1). However, the variation in methods used, and the previous absence of detailed HTS-based investigations, make direct comparisons difficult (Table 1).

**Table 1:** Comparison of selected *in vivo* and *in vitro* models of nisin activity.

***In vivo* or *in vitro* model**	**Nisin delivery**	**Highest nisin consumption per kg body weight per day**	**Nisin in feces**	**Microbiota**			**Rate of weight gain**	**References**
				**Location tested**	**Test method**	**Nisin effect**		
Mice	Oral	400 mg	Not tested	Not tested	Not tested	Not tested	Increased in one test group, no change in all other test groups	Shtenberg and Ignatev, 1970
Mice	Oral	161 mg (starch dough diet), 54 mg (starch gel diet)	Yes	Feces	16S rRNA MiSeq sequencing	Yes	No change	This paper
Mice	Oral	Not available	Not tested	Not tested	Not tested	Not tested	No change	de Pablo et al., 1999
Mice	Intraperitoneal injection	Not available	Not tested	Feces	Denaturing gradient gel electrophoresis (DGGE) of PCR-amplified 16S rDNA	Yes	Not tested	van Staden et al., 2011
Rats	Oral	225 mg (males), 239 mg (females)	Not tested	Not tested	Not tested	Not tested	No change	Hagiwara et al., 2010
Rats	Oral	174 mg^*^	Yes	Feces	Plating on selective media and denaturing gradient gel electrophoresis (DGGE) of PCR-amplified 16S rDNA	No	Not tested	Bernbom et al., 2006
Rats	Oral	50 mg	Not tested	Not tested	Not tested	Not tested	No change	Reddy et al., 2011
Rats	Oral	50 mg	Not tested	Not tested	Not tested	Not tested	No change	Gupta et al., 2008
Rats	Oral	10 mg	Not tested	Not tested	Not tested	Not tested	No change	Reddy et al., 2004
Quails	Oral	52 mg	Not tested	Not tested	Not tested	Not tested	No change	Ozdogan and Ustundag, 2015
Chickens	Oral	10 mg	Not tested	Ileum	DAPI staining and fluorescent *in situ* hybridization (FISH)	Yes	Increased	Józefiak et al., 2013
Chickens	Oral	10 mg	Not tested	Ileum	DAPI staining and fluorescent *in situ* hybridization (FISH)	Yes	Increased	Kieronczyk et al., 2016
Rabbits	Oral	20 μg	Not tested	Feces	Plating on selective media	Yes	Increased	Lauková et al., 2014
Fermentation vessel (bovine rumen)	Not relevant	Not relevant	Not relevant	Not relevant	16S RNA MiSeq sequencing	Yes	Not relevant	Shen et al., 2017
Fermentation vessel (human colon)	Not relevant	Not relevant	Not relevant	Not relevant	q-PCR coupled to propidium monoazide treatment	Yes	Not relevant	Le Lay et al., 2015

* *Extrapolated based on standard (Lillie et al., 1996) weight for rats of that age and breed*.

Starch is the primary carbohydrate source in the adult western diet (Sibley, 2004). Starch is comprised of the carbohydrate polymers amylose and amylopectin, and in plants these are arranged into semi-crystalline starch granules, which are of 0.1–200 μm in diameter. When “raw” starch granules are suspended in water and heated, the amylose, and amylopectin disassociate, with the granules leaching amylose and absorbing water causing them to swell and ultimately dissipate. When the solution is subsequently cooled, the amylose and amylopectin re-associate, turning the solution into a starch gel, with the gel strength primarily determined by amylose content. These two stages are referred to as gelatinization and retrogradation (Alcázar-Alay and Meireles, 2015; Wang et al., 2015). Many types of food processing, including cooking, can cause starch to undergo gelatinization and retrogradation (Delcour et al., 2010) with co-present substances becoming entrapped in the resulting starch gel (Forssell, 2004).

The portion of starch that resists digestion in the small intestine is termed “resistant starch” and varies between starch source and type. In the case of the type of starch used in this study (70% amylose starch from maize), the resistant starch content has been reported as 46% on a w/w basis (McCleary et al., 2002). Starch that is resistant due to its granular nature is classified as type 2 resistant starch (RS2), whereas starch that is resistant due to retrogradation is classified as type 3 resistant starch (RS3) (Sajilata et al., 2006). Due to the capacity of the resistant starch portion of a starch to resist digestion in the upper GIT and subsequently be fermented by colonic bacteria, starch based systems have been proposed for the colonic delivery of drugs and bioactive materials; these systems frequently use ethyl cellulose as a binder and are frequently produced through spray coating (Milojevic et al., 1996; Dimantov et al., 2004; Desai, 2005; Wilson and Basit, 2005; Freire et al., 2010; Pu et al., 2011; Situ et al., 2014; Recife et al., 2017).

The aim of this study was to determine the effect, *in vivo*, of orally consumed nisin on the lower GIT microbiota (as determined by 16S rRNA HTS of fecal samples; Suzuki and Nachman, 2016) when nisin was incorporated into two different starch based matrices; a dough based on raw starch (RS2) and a gel based on starch that had undergone gelatinization and retrogradation (RS3). Additionally the potential of the starch matrices themselves to impact on the microbiota was examined.

## Materials and methods

### Reagents

High amylose corn starch (HACS) was obtained from Sigma Aldrich (S4180, Sigma Aldrich, Arklow, Ireland). Dextrose equivalent 12 maltodextrin (DE12 MD) was obtained from Roquette (Glucidex® 12, Roquette, Corby, UK). All other reagents were from Sigma Aldrich (Arklow, Ireland) unless otherwise specified.

### Preparation of Nisin

The nisin A preparation used in this study was Nisaplin® (DuPont, Beaminster, UK). This preparation was concentrated by salting out as previously described (Gough et al., 2017a). This resulted in a 57.7% nisin preparation which will subsequently be referred to in the text as enriched nisin.

### Preparation of test diet pellets

Starch gels were prepared with and without nisin as follows. Starch gels with nisin were composed of 1% (w/w) enriched nisin, 44% (w/w) HACS, and 55% (w/w) dilute HCl, with a final pH of 3. Starch gels without nisin were composed of 45% (w/w) HACS and 55% (w/w) dilute HCl, with a final pH of 3. The suspensions were split into 10 mL aliquots, heated at 115°C for 15 min and subsequently incubated at 4°C for a minimum of 16 h to ensure thorough retrogradation. Starch dough was prepared with and without nisin as follows. The starch dough balls with nisin comprised 1% (w/w) enriched nisin, 51.5% (w/w) HACS, 22.5% DE12 MD, and 25% (w/w) dilute HCl. The starch dough balls without nisin contained 52.5% (w/w) HACS, 22.5% DE12 MD, and 25% (w/w) dilute HCl. For the preparation of the nisin containing starch dough balls, the dilute HCL and enriched nisin (at pH 3) were heated at 115°C for 15 min and allowed cool to room temperature before addition to the rest of the ingredients, to ensure that the treatment of the nisin in the starch dough was comparable with that of the nisin in the starch gel. All the components of the starch dough balls were then mixed in a laminar flow cabinet. Each starch dough ball was thoroughly kneaded to achieve homogeneity and firmness. The starch dough balls were stored at 4°C until use.

### Feeding schedule and sample collection

This study was carried out in accordance with European Directive 2010/63/EU. The protocol was approved by the University College Cork Animal Experimentation Ethics Committee (2011/005). Male C57BL/6JOlaHsd mice aged 3–4 weeks (Envigo, Alconbury, UK) were group housed (5 per cage) and were maintained in a 12:12 h light-dark cycle. During the initial 10 day acclimatization period, the mice were fed a standard nutritionally complete low-fat rodent diet (D12450B, Research Diets, New Brunswick, New Jersey, US); this diet is henceforth referred to as the nutritionally complete (NC) diet. Subsequently weight matched mice were assigned to receive the following test diets: starch dough (SD), starch dough containing nisin (SD-N), starch gel (SG), and starch gel containing nisin (SG-N) (*n* = 10 per test diet).

An overview of the feeding schedule is shown in Table 2. The feeding schedule involved initially switching the NC diets with the test diets for 2 h per day for 3 days and this was gradually increased to 8 h per day over the period of the trial as described in Table 2.The test diets were introduced gradually to acclimatize the animals to eating the starch based diets. The exposure to the NC diets thus decreased from 22 to 16 h per day over the period of the trial. The test diets were replaced every 4 days to ensure the freshness of the diet pellets. As mice are nocturnal animals and the cage room was on a 12:12 h light-dark cycle, the food hoppers were switched to test diets at the beginning of the dark cycle (18:00). The food hoppers were weighed throughout the trial as described in Table 2 and additional food hoppers in empty cages were used as controls to measure the impact of diet pellet drying on diet pellet weight. The hoppers were loaded with sufficient pellets of the test and NC diets to ensure that a sufficient quantity of test/NC diet was provided to the mice for *ad libitum* consumption at all times.

**Table 2:** Feeding schedule and days of fecal pellet collection and mouse and food hopper weighing.

**Day**	**1**	**2**	**3**	**4**	**5**	**6**	**7**	**8**	**9**	**10**	**11**	**12**	**13**	**14**	**15**	**16**
Fecal pellet collection	✓				✓				✓			✓			✓	
Mice weighed	✓	✓		✓				✓			✓					✓
Food hopper weighed	✓	✓		✓	✓	✓	✓	✓	✓	✓	✓	✓	✓	✓	✓	✓
Hours on test diet	2	2	2	4	4	4	4	4	6	6	6	8	8	8	8	n/a
Hours on nutritionally complete diet	22	22	22	20	20	20	20	20	18	18	18	16	16	16	16	n/a

The mice were weighed and fecal pellets collected during the course of the experiment as outlined in Table 2. At these time points fecal pellets were obtained from each mouse and stored at −80°C individually for 16S RNA sequencing. For MALDI TOF mass spectroscopy, HPLC and activity assays composite fecal samples were obtained by pooling the fecal pellets by cage at each time point. To limit contamination of the samples, the fecal pellets were collected directly from the mice and not from the bedding.

### DNA extraction, amplification, and sequencing

DNA was extracted from fecal pellets using a QIAamp® Fast DNA Stool Kit (Qiagen, Crawley, UK) according to the manufacturer's instructions with some modifications. To increase DNA yield, after the addition of InhibitEX buffer, bead beating (3 min × 2) and an incubation at 95°C for 5 min, were performed. The samples were quantified using a Qubit® dsDNA High Sensitivity Assay Kit (Fisher Scientific, Dublin, Ireland) in conjunction with a Qubit® 2.0 fluorometer (Invitrogen, Paisley, UK). The initial amplification PCRs were performed as outlined in the Illumina 16S Metagenomic Sequencing Library Preparation Guide (Illumina, Saffron Walden, UK) with the following alterations; 30 amplification cycles were used and the amplification PCRs were each performed in a total volume of 60 μL which contained 25 ng DNA and 1 μL of each primer at a 10 μM concentration. The subsequent clean up using the AMPure® XP purification system (Labplan, Dublin, Ireland) was scaled up appropriately to account for the greater volume. The index PCRs and subsequent AMPure® XP clean up were as outlined in the Illumina protocol. The samples were quantified using the Qubit® procedure and the concentrations normalized to 20 nM and pooled as per the Illumina protocol. The pooled sample (100 μL) was purified using AMPure® XP beads and the sample eluted using 50 μL of a 10 mM Tris solution. The pooled sample was quantified using the Qubit® procedure and sample quality was determined using an Agilent 2100 Bioanalyzer (Agilent, Cork, Ireland). The pooled sample was denatured and sequenced using a 500 cycle v2 kit on the MiSeq™ sequencing platform (Illumina, Saffron Walden, UK) following protocols outlined by Illumina at the Teagasc Sequencing Centre, Moorepark.

### Bioinformatics analysis

Sequences were filtered on the basis of quality (removal of low quality nucleotides at the 3′ end) and length (removal of sequences with < 200 nt) with PRINSEQ (Schmieder and Edwards, 2011) and joined using fastq-join (Aronesty, 2011). The sequences were clustered with 97% identity level (calculated at the operational taxonomic unit; OTUs) using closed-reference USEARCH v7.0 algorithm (Edgar, 2010) against the Ribosomal Database Project (Wang et al., 2007). Alpha and beta-diversity was determined using QIIME (Caporaso et al., 2010). The results of principal coordinates analysis (PCoA) of the beta-diversity when it was calculated using distance matrices built from unweighted UniFrac distances, were visualized using EMPeror (Vázquez-Baeza et al., 2013).

### Preparation of fecal pellets for detection of Nisin

To detect nisin in the fecal pellets, the nisin was extracted from the pellets as described by Rea et al. (2014) with minor modifications as follows: composite fecal samples were suspended in 1 mL of 0.1% TFA and 70% IPA, vortexed thoroughly and allowed to stand at room temperature for 30 min and centrifuged for 5 min at 16,000 × *g* and the supernatant retained. The centrifugation step was repeated a further three times with the supernatant retained each time. In order to bring the IPA content of the samples to <7%, IPA was removed using a Centrivap Console (Labconco, Kansas City, US) and the samples were then restored to their original volumes using 0.1% TFA.

### Reversed phase—high performance liquid chromatography (RP-HPLC)

RP-HPLC was carried out on a Jupiter, 5 μm, C18, 300 Å, 250 × 4.6 mm column from Phenomenex (Macclesfield, UK) with an acetonitrile (Thermo Fisher Scientific, Dublin, Ireland) gradient as described previously (Gough et al., 2017a).

### Matrix-assisted laser desorption/ionization time of flight mass spectroscopy (MALDI TOF MS)

The molecular mass of the HPLC fraction corresponding to the nisin peak was determined using MALDI TOF MS using an Axima TOF^2^ (Shimadzu Biotech, Kyoto, Japan) as previously described (Field et al., 2012).

### Activity assay

Antibacterial activity was estimated by agar diffusion activity assays (Ryan et al., 1996) in agar plates seeded with *L. lactis* subsp. *cremoris* HP as described previously (Gough et al., 2017a). Nisin was extracted from the fecal pellets as described above and Tween® 80 was added to a final concentration of 1% to prevent nonspecific adsorption of the nisin. The samples were dispensed into the wells of the seeded agar in 50 μL aliquots and the plates incubated overnight at 30°C. Antibacterial activity resulted in zones of inhibition surrounding the wells. Nisin was quantified based on a published method (Bernbom et al., 2006) by plotting the area of the zone of inhibition against the log of the nisin concentration of a serial dilution of Nisaplin® that was suspended in an equivalent solution to the samples (6% IPA, 0.1% TFA, 1% Tween® 80), to generate a linear standard curve.

### Statistical analysis

Data was tested for normality of distribution using the Shapiro–Wilk test. For comparing two groups Student's *t*-test or Mann–Whitney *U*-test were used as appropriate and for comparison of multiple groups one-way ANOVA or Kruskal–Wallis test were used as appropriate, additionally analysis of beta diversity was performed using the Adonis function in the R package Vegan (Oksanen et al., 2015). Analysis of the bioinformatics data was performed using the R statistical package (R Core Team, 2015) and all other analysis was performed using the SigmaStat software (Systat Software, San Jose, US). Results are expressed as mean ± standard error.

## Results

### Quantity of diets consumed and effect on weight gain

**Figure 1:**
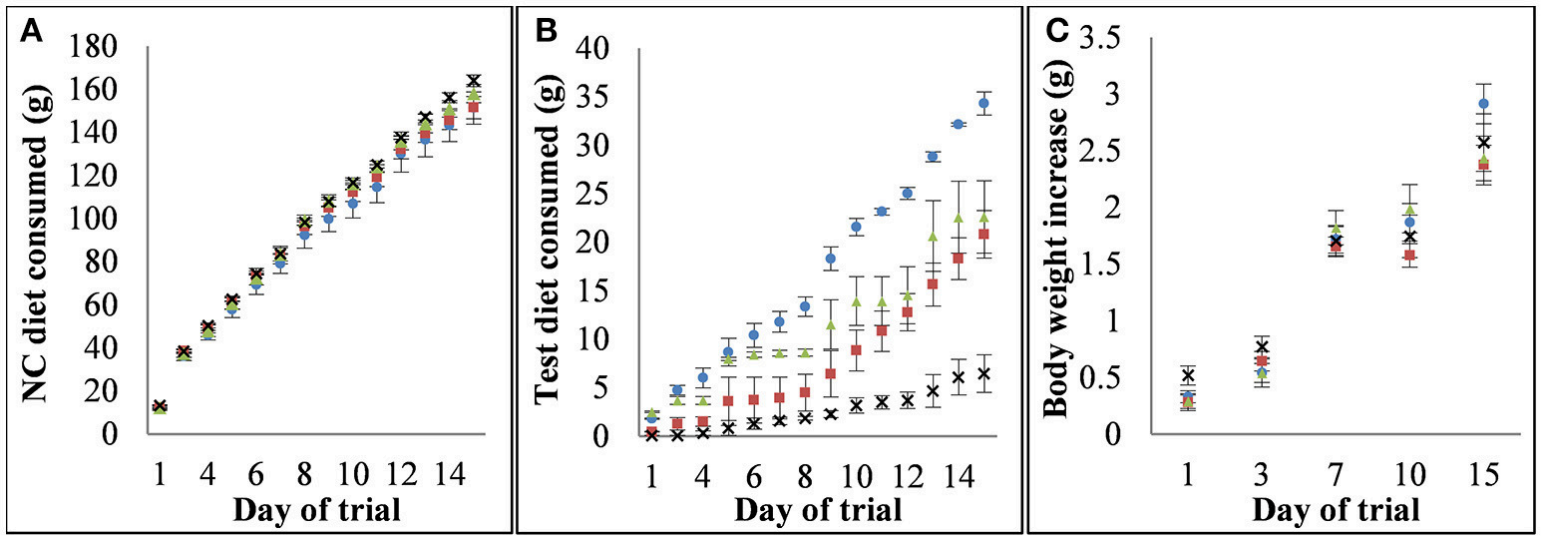
Consumption of each diet and the relationship between diets and weight gain. **(A)** Cumulative consumption of nutritionally complete (NC) partner diets for each diet group, **(B)** cumulative consumption of test diets for each diet group, **(C)** cumulative weight gain for each diet group. Diet groups are defined by their test diet as follows: blue circle - starch dough (SD), red square - starch dough containing nisin (SD-N), green triangle - starch gel (SG), black cross - starch gel containing nisin (SG-N).

The cumulative consumption of the NC and test diets and resultant body weight gain are shown in Figure 1. There were no significant differences in body weight gain or in NC diet consumption between diet groups over the trial period with three exceptions, each of which occurred only at a single measurement time point; the consumption of the NC partner diet for SD and SG-N in the 6 h consumption period was significantly different (*p* = 0.02, *n* = 6), the weight gain for the mice on the SG and SG-N diets from days 4 to 7 of the trial was significantly different (*p* = 0.02, *n* = 10) and the weight gain for the mice on the SD and SG diets from days 11 to 15 of the trial was significantly different (*p* = 0.0004, *n* = 10). The total consumption per cage of the SD-N and SG-N test diets was 20.8 ± 2.5 and 6.5 ± 2.0 g, respectively, and the daily consumption of these diets were significantly different during the 6 h (*p* = 0.00007, *n* = 6) and 8 h (*p* = 0.00003, *n* = 8) consumption period. The total nisin consumption per cage over the course of the trial was 144 ± 14 and 52 ± 11 mg for the SD-N and SG-N diet groups, respectively, and the daily consumption of the nisin portion of those diets were also significantly different during the 6 h (*p* = 0.0003, *n* = 6) and 8 h (*p* = 0.00002, *n* = 8) consumption period. The average nisin consumption per day per cage during the 8 h consumption period was 17 ± 1 and 6 ± 2 mg for the SD-N and SG-N diets groups, respectively. Therefore, there was approximately a three-fold greater consumption of nisin by mice on the SD-N diet compared to mice on the SG-N diet. For SD-N compared to SD, SG-N compared to SG and SD compared to SG there were no statistically significant differences during the 6 h (*p* = 0.134, 0.101, and 0.217, respectively, *n* = 6) and 8 h (*p* = 0.507, 0.442, and 0.54, *n* = 8) consumption periods (days 9–15 of the trial).

### Identification and quantification of intact Nisin and Nisin fragments in the feces

**Figure 2:**
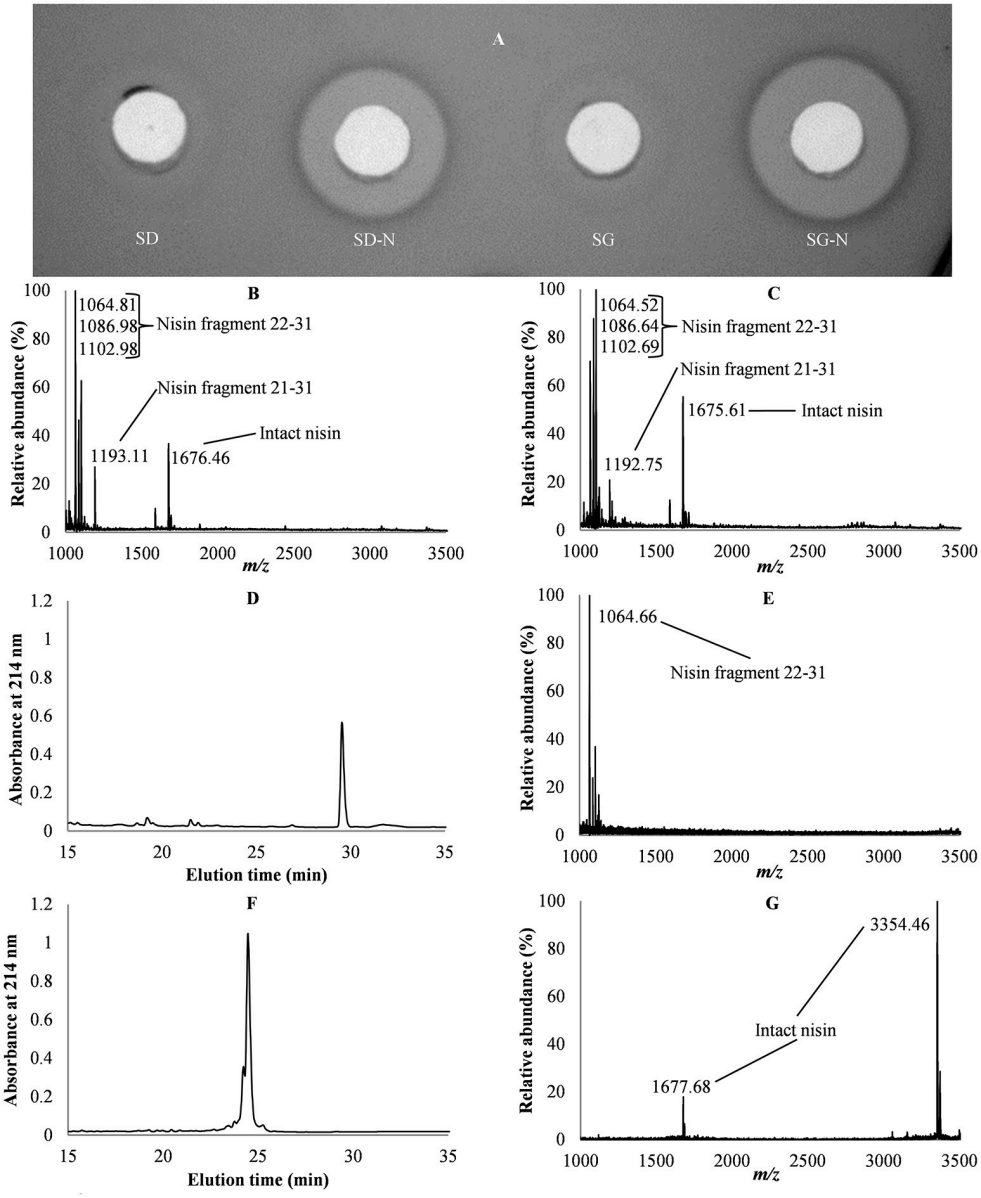
Analysis of fecal pellets of mice consuming starch dough (SD), starch dough containing nisin (SD-N), starch gel (SG), and starch gel containing nisin (SG-N) diets. Activity assay of fecal pellets of mice consuming SD, SD-N, SG, and SG-N diets **(A)**. Mass spectroscopy of fecal pellets from mice consuming SD-N **(B)** and SG-N diets **(C)**. RP-HPLC chromatogram of fecal pellets from mice consuming SG-N **(D)** and mass spectroscopy of the elution peak **(E)**. RP-HPLC chromatogram of intact nisin **(F)** and mass spectroscopy of the elution peak **(G)**.

The activity assays of the fecal pellets from mice consuming SD, SD-N, SG, and SG-N diets (Figure 2A) showed antibacterial activity in feces from mice that consumed the SD-N and SG-N diets. MALDI TOF MS was performed on fecal pellets to determine their intact nisin and nisin fragment composition (Figures 2B,C). Their primary nisin components were then determined by RP-HPLC in conjunction with MALDI TOF MS (Figures 2D,E). For comparison purposes intact nisin was also analyzed by RP-HPLC in conjunction with MALDI TOF MS (Figures 2F,G).

MALDI TOF MS of the fecal pellets of mice on the SD-N (Figure 2B) and SG-N diets (Figure 2C) showed masses that correlated with intact nisin and nisin fragments 22–31 (i.e., corresponding to amino acids 22–31 of intact nisin) and 21–31; these nisin fragments are the products of the digestion of nisin and have predicted molecular masses of 1063.47 and 1195.44 Da, respectively (Slootweg et al., 2013). Versions of nisin fragment 22–31 with a Na adduct ion (+22 Da) and a K adduct ion (+38 Da) were also detected. Intact nisin, extracted from the fecal pellets, was seen in its doubly charged form at 1676.46 and 1675.61 Da for the SD-N diet and for the SG-N, respectively.

RP-HPLC of the fecal pellets of mice on the SG-N diet showed a single dominant peak (Figure 2D) that eluted at 41% acetonitrile and MALDI TOF MS of this peak revealed it to be nisin fragment 22–31 (Figure 2E). A similar result was obtained for the fecal pellets of mice on the SD-N diet (result not shown). Therefore, the primary nisin component of the feces was fragment 22–31, as opposed to intact nisin.

Intact nisin normally elutes from a RP-HPLC at 36% acetonitrile (Figure 2F) and subsequent MALDI TOF MS of this elution peak shows both singly (3354.46 Da) and doubly (1677.68 Da) charged intact nisin (Figure 2G). However, while no intact nisin was detected by HPLC, antibacterial activity was detected in the feces of those groups fed the SD-N and SG-N diets (Figure 2A). This would suggest that the nisin concentration in the fecal pellets was below the level of detection by HPLC.

Quantifying the intact nisin in the feces at the final time point based on antibacterial activity showed significantly more (*p* = 0.031, *n* = 3) nisin in the feces of the group fed SG-N (1.7 ± 0.2 ng/mg) compared to the groups fed SD-N (0.8 ± 0.1 ng/mg), despite the fact that less nisin was consumed by the group fed the SG-N diet, which would indicate that more intact nisin reached the lower GIT in SG-N-fed mice. Therefore, at the final time point (8 h test diet period), despite the significantly (*p* = 0.00002, *n* = 8) greater nisin consumption of the mice on the SD-N diets, there was significantly (*p* = 0.031, *n* = 3) greater nisin in the feces from consumption of the SG-N diets.

### HTS-based analysis of microbiota

**Figure 3:**
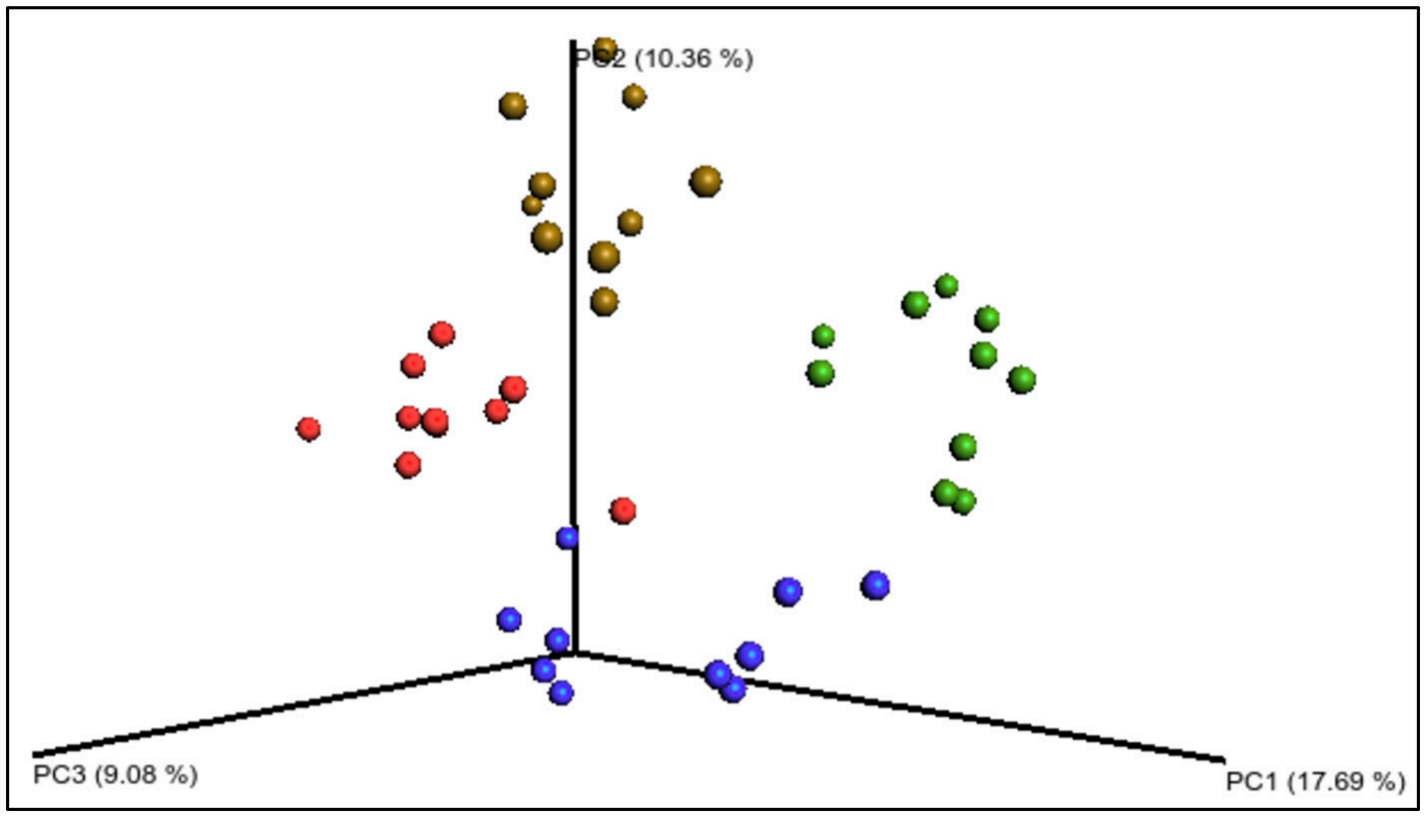
Principal coordinates analysis PCoA of the unweighted UniFrac distances of the 168 sequencing data. The four diet groups are represented by colored circles: blue—starch dough (SD), green—starch dough containing nisin (SD-N), red—starch gel (SG), brown—starch gel containing nisin (SG-N). The groups are significantly different (*p* = 0.001, *n* = 10).

Following total metagenomic DNA extraction from the fecal pellets from day 15, 16S rRNA gene amplicons (V3–V4 region) were generated and sequenced using the Illumina MiSeq™ platform. The mean number of sequence reads and alpha diversity indices for each diet group are shown in Table 3. There were no statistical differences in the alpha diversity indices: Observed operational taxonomic units (unique operational taxonomic units), Chao1 (richness), ACE (richness), Simpson (richness and evenness), and Shannon (richness and evenness), between the diet groups (Table 3). However, when the beta diversity was calculated using distance matrices built from unweighted UniFrac distances and the PCoA results visualized using EMPeror (Vázquez-Baeza et al., 2013), the four treatment groups formed distinct clusters based on diet (Figure 3), which were significantly different (*p* = 0.001, *n* = 10).

**Table 3:** Mean sequence reads and alpha diversity indices for starch dough (SD), starch dough containing nisin (SD-N), starch gel (SG), and starch gel containing nisin (SG-N) diet groups (mean ± standard error, *n* = 10).

	**SD**	**SD-N**	**SG**	**SG-N**
Sequence reads	43,465 (±7,276)	52,311 (±4,629)	39,848 (±3,909)	42,903 (±4,969)
Observed operational taxonomic units	267 (±19)	300 (±12)	246(±11)	296 (±22)
Chao1	277 (±18)	309 (±12)	254(±12)	303 (±22)
ACE	279 (±18)	310 (±12)	256(±11)	304 (±22)
Shannon	3.58 (±0.03)	3.58 (±0.05)	3.57(±0.04)	3.69 (±0.10)
Simpson	0.947 (±0.002)	0.941 (±0.005)	0.947(±0.003)	0.940 (±0.007)
Inverse simpson	19.1 (±0.8)	17.8 (±1.2)	19.4(±1.0)	18.7 (±2.2)

**Figure 4:**
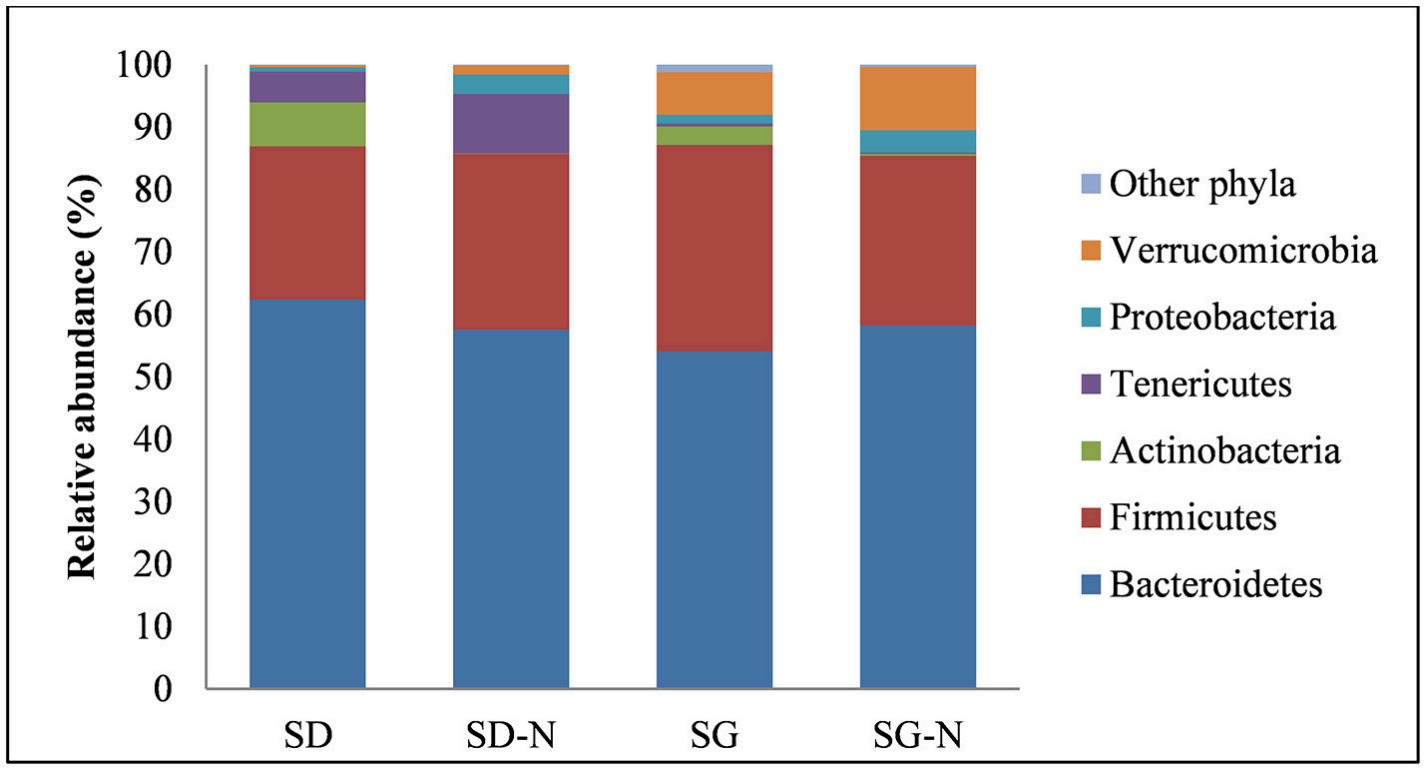
Relative abundance at phylum level with respect to each diet. Diet groups are as follows: starch dough (SD), starch dough containing nisin (SD-N), starch gel (SG), and starch gel containing nisin (SG-N) diet (*n* = 10).

Sequence analysis revealed that the microbiota were primarily comprised of six phyla and that *Bacteroidetes* and *Firmicutes* were the dominant phyla showing a relative abundance of 54–62 and 25–33%, respectively. There were no significant differences between the relative abundance of *Bacteroidetes* and *Firmicutes* across the diet groups (Figure 4), however there were significant differences (*p* < 0.001, *n* = 10) in the relative abundance between diet groups in the phyla *Actinobacteria, Tenericutes*, and *Verrucomicrobia* (Figure 4).

**Figure 5:**
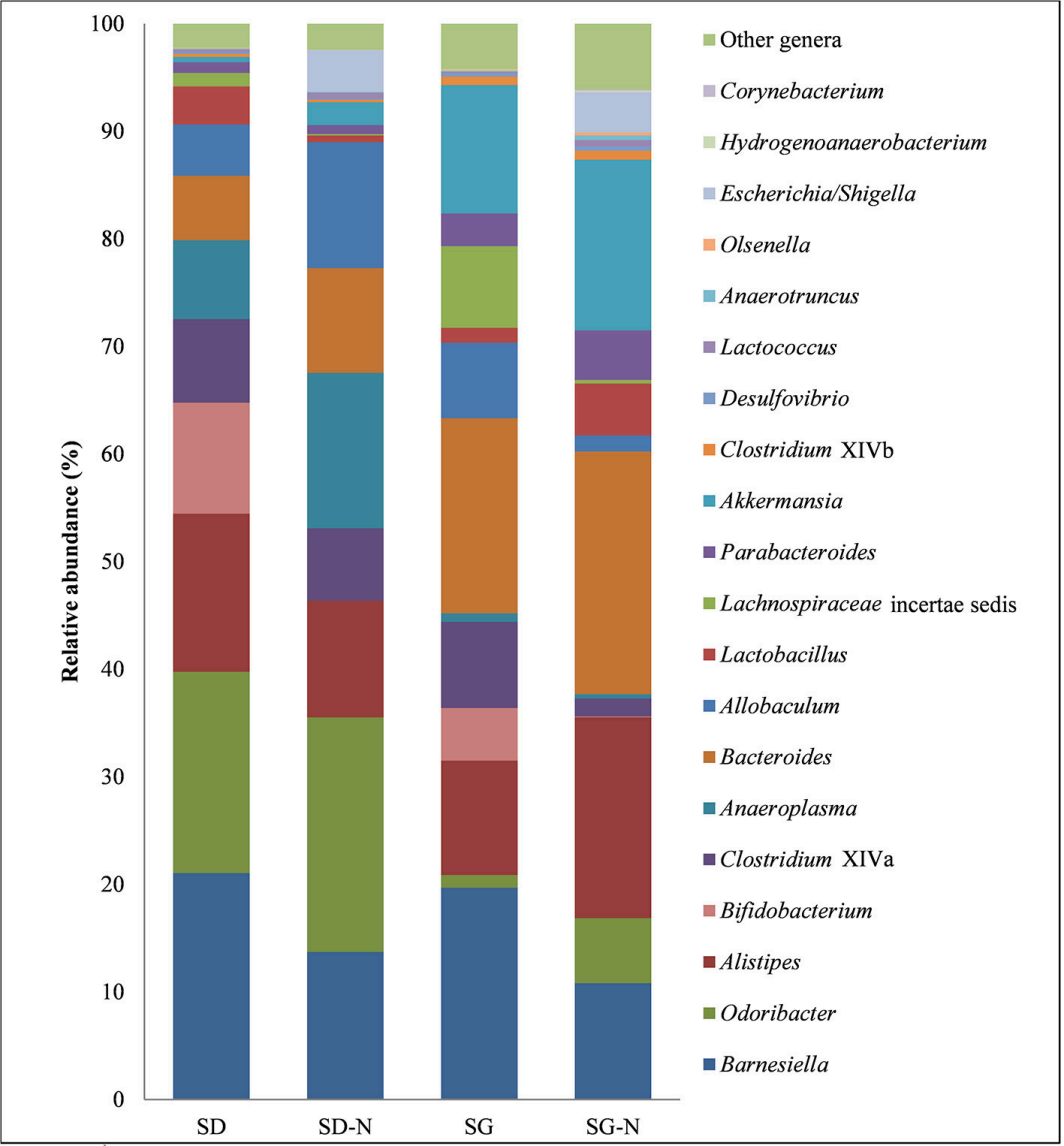
Relative abundance at genus level with respect to each diet. Diet group are as follows: starch dough (SD), starch dough containing nisin (SD-N), starch gel (SG), and starch gel containing nisin (SG-N) diet (*n* = 10).

A number of statistical differences were found at genus level between the different dietary groups (Figure 5 and Table 4). The mice fed the SG-N diet had significantly lower relative abundance of the genera *Allobaculum, Bifidobacterium, Lachnospiracea* incertae sedis, and *Clostridium* cluster XIVa and significantly higher relative abundance of the genera *Escherichia/Shigella, Lactococcus*, and *Corynebacterium* compared to the mice fed the SG diet (*p* < 0.001, *n* = 10). These changes were reflected at the corresponding family level. However, there was also a significantly higher (*p* = 0.0005, *n* = 10) relative abundance of the family *Ruminococcaceae* (Table 4) in mice fed the SG-N diet that did not correspond to a significant increase of any genus related to the *Ruminococcaceae* family. This likely reflects the combined increases (not individually statistically significant) in the proportions of the genera *Anaerotruncus* and *Hydrogenoanaerobacterium*, i.e., members of the *Ruminococcaceae* family, in mice that consumed the SG-N diet. Relative to the SD diet, the SD-N diet significantly (*p* < 0.001, *n* = 10) affected the relative abundance of only three genera; i.e., *Lactobacillus* and *Bifidobacterium*, which were lower, and *Escherichia/Shigella*, which were higher (Table 4).

**Table 4:** Bacterial taxa whose relative abundance was significantly different between diet groups.

	**SD**	**SD-N**	**SG**	**SG-N**
**SIGNIFICANT AT GENUS LEVEL**				
*Akkermansia*	0.499 (±0.111)a	2.120 (±1.077)	11.943 (±2.369)a	15.879 (±4.789)
*Allobaculum*	4.764 (±0.827)	11.690 (±2.107)b	7.018 (±1.245)d	1.504 (±0.528)bd
*Anaeroplasma*	7.307 (±1.905)a	14.442 (±2.667)b	0.799 (±0.180)a	0.385 (±0.174)b
*Bifidobacterium*	10.317 (±0.902)ac	0.005 (±0.001)bc	4.894 (±0.602)ad	0.090 (±0.030)bd
*Clostridium* cluster XIVa	7.777 (±1.044)	6.731 (±0.577)b	8.003 (±1.261)d	1.685 (±0.646)bd
*Clostridium* cluster XIVb	0.289 (±0.095)	0.226 (±0.045)b	0.777 (±0.117)	0.831 (±0.145)b
*Corynebacterium*	0.007 (±0.002)	0.021 (±0.007)	0.007 (±0.003)d	0.091 (±0.018)d
*Desulfovibrio*	0.222 (±0.058)	0.119 (±0.031)b	0.350 (±0.076)	0.415 (±0.058)b
*Escherichia/Shigella*	0.009 (±0.005)c	3.860 (±1.971)c	0.011 (±0.002)d	3.771 (±1.795)d
*Lachnospiraceae* incertae sedis	1.248 (±0.375)a	0.126 (±0.091)	7.579 (±1.410)ad	0.336 (±0.281)d
*Lactobacillus*	3.536 (±0.470)c	0.643 (±0.330)c	1.378 (±0.351)	4.828 (±3.140)
*Lactococcus*	0.168 (±0.024)	0.533 (±0.080)	0.095 (±0.014)d	0.580 (±0.143)d
*Odoribacter*	18.711 (±2.515)a	21.817 (±1.322)b	1.164 (±0.186)a	6.058 (±2.205)b
*Parabacteroides*	0.996 (±0.160)a	0.835 (±0.108)	3.042 (±0.662)a	4.597 (±1.200)
**NOT SIGNIFICANT AT GENUS LEVEL BUT SIGNIFICANT AT FAMILY LEVEL**				
*Ruminococcaceae*	2.285 (±0.468)	3.296 (±0.420)	2.157 (±0.273)d	7.759 (±0.684)d
Unclassified *Ruminococcaceae*	1.519 (±0.356)	2.228 (±0.259)	1.492 (±0.201)d	5.284 (±0.423)d

*Diet groups are as follows: starch dough (SD), starch dough containing nisin (SD-N), starch gel (SG), and starch gel containing nisin (SG-N). The relative abundance of each bacterial taxon is expressed as mean ± standard error. The same letter after a pair of values in a single row indicates these values are significantly different (p < 0.001, n = 10): (a) SD compared to SG, (b) SD-N compared to SG-N, (c) SD compared to SD-N, and (d) SG compared to SG-N*.

There were also differences between the diet groups when compared on the basis of starch matrix. The relative abundance of the genera *Anaeroplasma, Bifidobacterium*, and *Odoribacter* were significantly (*p* < 0.001, *n* = 10) greater in the mice fed the SD diet compared to the SG diet, whereas the relative abundance of the genera *Akkermansia, Lachnospiracea* incertae sedis, and *Parabacteroides* were all significantly (*p* < 0.001, *n* = 10) greater in the mice fed the SG diet relative to the SD diet (Figure 5 and Table 4). In addition, *Clostridium* cluster XIVb and *Desulfovibrio* had greater relative abundance in the mice fed the SG and SG-N diets compared to the mice fed the SD and SD-N diets (Table 4) and this was significantly different (*p* < 0.001, *n* = 10) for the SG-N diet group compared to the SD-N diet group.

## Discussion

The aim of this study was to determine if orally ingested nisin could be delivered to the lower GIT in two different starch matrices and subsequently impact on the lower GIT microbiota. Additionally, it was examined whether the type of starch itself could modulate the lower GIT microbiota.

To the authors' knowledge (Table 1) the only study that has examined the effect of orally ingested nisin on the rodent microbiota is the study on rats by Bernbom et al. (2006), in which the highest amount of nisin consumed was 174 mg nisin per kg body weight per day and, while nisin was detected in the feces, no changes in the microbiota were detected which may be due to the sensitivity of the molecular methods used in that study. In this study it was hoped that using a 16s HTS approach and similar levels of nisin as described by Bernbom et al. (2006), it would be possible to determine the impact of nisin on the microbiota. All test diets were increased at intervals over the trial to acclimatize the mice to consuming starch and nisin. At the 8 h consumption period the mice consumed 161 and 54 mg nisin per kg body weight per day for the SD-N and SG-N diets, respectively. To limit the stress on the mice they were allowed unrestricted access to a diet within a given consumption period, however this approach limited a more precise matching of the amount fed to the Bernbom et al. (2006) study.

Numerous studies have shown that nisin is susceptible to digestion by the enzymes in the upper GIT and a previous study by our group using the *in vitro* INFOGEST digestion model for the human GIT, detected nisin fragments corresponding to the N-terminus of nisin (amino acids 1–11, 1–12, 1–20, 1–21, 1–29, and 1–32) post digestion, while no intact nisin was detected (Gough et al., 2017b). In this study, low levels of biologically active nisin (ng/mg of feces) were detected in the feces of mice fed SG-N and SD-N, but, in contrast to the *in vitro* study, the primary nisin component of the feces was fragment 22–31, which is not biologically active as the N-terminus is required for nisin activity (Hsu et al., 2004). It is also notable that the fragments produced by the *in vivo* digestion had a significant portion of their C-terminal present whereas those produced by the *in vitro* digestion had a significant portion of their N-terminal present. These differences can most likely be attributed to species-related differences in digestive enzymes.

More nisin was detected in the fecal samples of the mice on the SG-N diet despite them having consumed less nisin than those on the SD-N diet; implying that more intact nisin reached the lower GIT on the SG-N diet and that the starch gel may have afforded some protection to the nisin from digestion in the upper GIT. To the authors' knowledge there are no reported studies of the *in vivo* effect of nisin on the gut microbiota using HTS techniques. The results of 16S rRNA compositional sequencing showed that alpha diversity indices for all diet groups were comparable to those seen in previous studies on fecal samples from C57BL/6J mice on low-fat diets (Allen et al., 2015; Javurek et al., 2017). Notably, however, beta diversity analysis showed that the murine microbiotas clustered together on the basis of diet. With respect to taxonomy, significant differences in the relative abundance between diet groups were observed for the phyla *Actinobacteria, Tenericutes*, and *Verrucomicrobia* (*p* < 0.001, *n* = 10). In each case a single genus, i.e., *Bifidobacterium, Anaeroplasma*, and *Akkermansia*, respectively, comprised the majority (>98%) of the genera detected belonging to these phyla.

There were differences between the cumulative consumption of SD compared to SG (Figure 1B), however as detailed above, those differences were not statistically significant for the 6 and 8 h consumption periods (days 9–15 of the trial). Resistant starch is known to effect satiety (Lockyer and Nugent, 2017). The hormones glucagon-like peptide 1 (GLP-1) and peptide tyrosine-tyrosine (PYY) which are involved in the regulation of satiety and glycemic response (D'Alessio, 2008) have been demonstrated to be elevated by resistant starch consumption in studies on mice (Zhou et al., 2008). Although to the authors knowledge while the relative ability of difference resistant starch types to effect satiety has not been elucidated, it has been shown that different types of resistant starch elicit significantly different glycemic responses (Haub et al., 2010). Therefore, it may be possible that the differences in consumption of the SD and SG are due to differences in the effect of RS2 and RS3 on satiety, however this was not investigated further in this study given our focus on the effects of nisin on the gut microbiota.

There were also differences between the cumulative consumption of SG-N compared to SG, and SD-N compared to SD (Figure 1B), however as detailed above, those differences were not statistically significant for the 6 and 8 h consumption periods (days 9–15 of the trial). This reduction in consumption is unlikely to be due to an effect of nisin on the microbiota as such a change in the microbiota would also affect the consumption of the NC diets and there were no statistically significant differences between the consumption of their respective partner NC diets throughout the trial (Figure 1A).While high protein diets have been shown to increase satiety, the level of protein in the nisin containing diets (~0.58%) is unlikely to have had an effect on satiety in this case (Batterham et al., 2006; Yu et al., 2009; Wiessing et al., 2015). However, it is possible that the palatability of nisin may have contributed to the reduced consumption of the nisin containing diets.

A limitation of this study is that the mice consumed different quantities of each of the test diets (Figure 1B). While this could have confounded the effect of nisin on the microbiota of the diet groups when SD is compared to SD-N and SG is compared to SG-N (Table 4), the changes in the microbiota, nonetheless, are consistent with the specific effect of nisin on these microorganisms. The difference in the amount of starch consumed and resistant starch type could have confounded the effects of the nisin. However, when comparing diets containing dough and gel (Table 4), of the eight genera that showed a significant difference (*p* < 0.001, *n* = 10) in relative abundance, only two genera were also significantly different (*p* < 0.001, *n* = 10) in relative abundance when SD-N was compared to SD and SG-N was compared to SG. Additionally, of these two genera, *Lachnospiraceae* incertae sedis was only significantly different (*p* < 0.001, *n* = 10) in relative abundance in SG-N compared to SG, while *Bifidobacterium* showed significantly lower (*p* < 0.001, *n* = 10) relative abundance in both the SD-N compared to SD and SG-N compared to SG. It has been reported previously that *Bifidobacterium* are particularly sensitive to nisin relative to other intestinal bacteria (Le Blay et al., 2007). Furthermore, nisin primarily targets Gram positive bacteria. Interestingly the genera that were significantly lower (*p* < 0.001, *n* = 10) in relative abundance in SD-N compared to SD and SG-N compared to SG (Table 4) were Gram positive or primarily Gram positive (*Clostridium* cluster XIVa), whereas the genera and family that significantly increased (*p* < 0.001, *n* = 10) in relative abundance in SD-N compared to SD and SG-N compared to SG (Table 4) were either Gram negative (*Escherichia/Shigella*), contained Gram negative members (*Ruminococcaceae*) or may have had nisin resistant mechanisms that are known to be present in some strains (*Lactococcus* and *Corynebacterium*) (Brenner et al., 2005; De Vos et al., 2009; Goodfellow et al., 2012; Zhou et al., 2014; Draper et al., 2015; Gharsallaoui et al., 2016). Taking these points together, we hypothesize that one reason for the differences in relative abundance between SD-N compared to SD and SG-N compared to SG is the presence or absence of nisin in the test diets.

Starch based doughs have been proposed for use for the oral delivery of drugs to laboratory rodents as a stress free alternative to oral gavage (Corbett et al., 2012). We observed in preliminary *in vitro* studies, that SD-N when placed in water rapidly dissociated releasing the nisin, whereas SG-N did not dissociate and nisin release was limited. Therefore, it is possible that the nisin would be released earlier and more rapidly from the SD-N than from the SG-N, which would in turn result in more of the nisin being digested in the upper GIT by the digestive enzymes secreted there and therefore impacting less on the microbiota in the lower GIT than nisin incorporated into the SG-N. While it is acknowledged that there are difficulties discerning the effect of the rate of release of the starch matrices from the effect of the level of consumption and resistant starch type, the compositional sequencing provides some evidence that nisin was released from the SD-N early in GIT transit and from the SG-N late in GIT transit. The relative abundance of *Lactobacillus*, which are primarily residents in the upper GIT that in turn transiently populate the lower GIT (Denev, 2006; Walter, 2008), was reduced in the SD-N fed group but were unaffected in the SG-N fed group, which may point to an earlier release in the upper GIT resulting in fewer lactobacilli reaching the colon. Additionally the SG-N diet affected the relative proportion of more than three times as many genera that are primarily resident in the lower GIT than were affected by the SD-N diet (when comparing both with their respective “starch only” controls); this indicates that the SG-N delivered more nisin to the lower GIT than the SD-N. Furthermore, despite there being approximately three-fold lower consumption of nisin by the mice on the SG-N diet compared to the mice on the SD-N diet, there was approximately twice as much nisin detected in the feces of the mice that consumed the SG-N diet compared to those that consumed the SD-N diet.

*Bifidobacterium* and *Escherichia/Shigella* were the only two genera significantly (*p* < 0.001, *n* = 10) different in both the SD-N and SG-N diet groups compared to the SD and SG diet groups. Bifidobacteria have been demonstrated to attenuate *Escherichia/Shigella* in several studies, including in mice (Gibson and Wang, 1994; Shu and Gill, 2001; Cheikhyoussef et al., 2007). It is possible that a nisin mediated reduction in the relative abundance of bifidobacteria allowed *Escherichia/Shigella* to increase in relative abundance; particularly as these were the only two genera resident in the lower GIT that were significantly different when the SD-N and SD diet groups were compared.

While it would be interesting to determine whether the changes in the microbiota seen in this study could occur at substantially lower levels of nisin consumption such as those found in food, a dose response study would be required to evaluate this. The current acceptable daily intake (ADI) is 1 mg nisin per kg body weight per day (Younes et al., 2017) while typical levels added to foods range from 2.5 to 25 mg/kg (Delves-Broughton, 2005).

Resistant starch is capable of modulating the microbiota in the lower GIT and its effect depends on the type of resistant starch (Bird et al., 2000; Martínez et al., 2010). Many of the genera whose relative abundance was significantly different (*p* < 0.001, *n* = 10) when compared on the basis of resistant starch type including *Akkermansia, Anaeroplasma, Bifidobacterium, Lachnospiracea, Odoribacter*, and *Parabacteroides* have positive health associations (Leahy et al., 2005; Noor et al., 2010; Kverka et al., 2011; Reeves et al., 2012; Vital et al., 2014; Zeng et al., 2015; Gómez-Gallego et al., 2016). Of particular interest was the alteration in the relative abundance of *Akkermansia* which has been described as a “next generation probiotic” (Cani and Van Hul, 2015) and is associated with numerous health benefits including treating type 2 diabetes, reducing the occurrence of autoimmune diseases and in weight management (Gómez-Gallego et al., 2016). *Akkermansia* in the murine gut is generally low (Schubert et al., 2015). Diets that include resistant starch have been shown previously to increase the relative abundance of *Akkermansia* (Tachon et al., 2013). There was less SG (RS3) consumed than SD (RS2) over the course of the study (Figure 1B), although as detailed above, this difference in consumption was not significant during the 6 or 8 h consumption periods (days 9–15 of the trial), however the relative abundance of *Akkermansia* was significantly (*p* = 0.0002, *n* = 10) greater in mice fed the SG (RS3) than the SD (RS2) diet (12 and 0.5% relative abundance, respectively). This may be attributable to the type of starch, however confirmation of this would require further investigation using NC diets incorporating the various starch types.

Overall, while it may be possible to attribute the differences in the microbiota between the diet groups to the effects of the diet components, it is important to highlight that these may not all be direct effects. The GIT microbiota is an interdependent community and the effect of a diet component on members of that network may promote other members that were not directly affected by the diet component (Willing et al., 2011; Scott et al., 2015).

Increased body weight gain due to nisin consumption has been demonstrated in previous studies involving chickens and rabbits (Table 1). However, in this study, no effect of nisin on body weight was observed, regardless of the matrix used for delivery. This is consistent with studies involving rats and quails and the majority of studies involving mice (Table 1).

## Conclusions

This study showed that by using a starch matrix, nisin can be delivered to the lower GIT and will impact on the lower GIT microbiota. All four diets altered the mouse microbiota differently, with the differences between the two nisin containing diets may be attributable to differences in how nisin was released and protected by the two starch matrices, while the differences between the starch matrices may be attributable to the type of resistant starch (type 2 and type 3) favoring the abundance of different bacterial taxa. It was particularly notable how the relative abundance of the probiotic *Akkermansia* differed between the two resistant starch diets however the difference in consumption between starch diets makes comparisons more difficult and this would need to be addressed in a further study. Despite greater consumption of the SD-N diet, the SG-N diet resulted in larger amounts of intact nisin in the feces and appeared to affect a greater number of lower GIT bacterial taxa. This highlights the importance of the matrix when studying the activity of a bioactive peptide either as a food additive or as a therapeutic for gastrointestinal pathogens. This study also demonstrated, in an *in vivo* model, the usefulness of resistant starch, particularly in a retrograded gel, for the colonic delivery of a bioactive peptide. This system may be of use for other heat stable peptides, including those with a narrower range of antimicrobial activity.

## Author contributions

RG, AB, SM, CH, RR, PC, KN, and MR contributed to the design of the experiments. RG, PO, FC, and KN performed the experiments. RG performed statistical analysis of the data. RC performed the bioinformatic analysis of the data. RG, RC, PO, CH, RR, KN, and MR contributed to the interpretation of the data. RG wrote the manuscript. RC, PO, FC, AB, SM, CH, RR, PC, KN, and MR revised the manuscript. All authors approved of the final manuscript being submitted and agree to be accountable for the work detailed in the submitted manuscript.

### Conflict of interest statement

The authors declare that the research was conducted in the absence of any commercial or financial relationships that could be construed as a potential conflict of interest. The reviewer GG and handling Editor declared their shared affiliation.

## References

[B1] Abee T. Delves-Broughton J. (2003). Bacteriocins - nisin, in Food Preservatives, 2nd Edn, eds Russell N. J. Gould G. W. (New York, NY: Kluwer Academic; Plenum Publishers), 146–178.

[B2] Alcázar-Alay S. C. Meireles M. A. A. (2015). Physicochemical properties, modifications and applications of starches from different botanical sources. Food Sci. Technol. 35, 215–236. 10.1590/1678-457X.6749

[B3] Allen J. M. Berg Miller M. E. Pence B. D. Whitlock K. Nehra V. Gaskins H. R.. (2015). Voluntary and forced exercise differentially alters the gut microbiome in C57BL/6J mice. J. Appl. Physiol. 118, 1059–1066. 10.1152/japplphysiol.01077.2014 25678701

[B4] Aronesty E. (2011). ea-utils: Command-line Tools for Processing Biological Sequencing Data. Available online at: https://github.com/ExpressionAnalysis/ea-utils

[B5] Batterham R. L. Heffron H. Kapoor S. Chivers J. E. Chandarana K. Herzog H.. (2006). Critical role for peptide YY in protein-mediated satiation and body-weight regulation. Cell Metabol. 4, 223–233. 10.1016/j.cmet.2006.08.001 16950139

[B6] Bernbom N. Licht T. R. Brogren C. H. Jelle B. Johansen A. H. Badiola I.. (2006). Effects of *Lactococcus lactis* on composition of intestinal microbiota: role of nisin. Appl. Environ. Microbiol. 72, 239–244. 10.1128/AEM.72.1.239-244.2006 16391049 PMC1352298

[B7] Bird A. R. Brown I. L. Topping D. L. (2000). Starches, resistant starches, the gut microflora and human health. Curr. Issues Intest. Microbiol. 1, 25–37. 11709851

[B8] Brenner D. J. Krieg N. R. Staley J. T. Garrity G. M. (eds.). (2005). Bergey's Manual of Systematic Bacteriology. New York, NY: Springer.

[B9] Cani P. D. Van Hul M. (2015). Novel opportunities for next-generation probiotics targeting metabolic syndrome. Curr. Opin. Biotechnol. 32, 21–27. 10.1016/j.copbio.2014.10.006 25448228

[B10] Caporaso J. G. Kuczynski J. Stombaugh J. Bittinger K. Bushman F. D. Costello E. K.. (2010). QIIME allows analysis of high-throughput community sequencing data. Nat. Methods 7, 335–336. 10.1038/nmeth.f.303 20383131 PMC3156573

[B11] Chan W. C. Leyland M. Clark J. Dodd H. M. Lian L. Y. Gasson M. J.. (1996). Structure-activity relationships in the peptide antibiotic nisin: antibacterial activity of fragments of nisin. FEBS Lett. 390, 129–132. 10.1016/0014-5793(96)00638-2 8706842

[B12] Cheikhyoussef A. Pogori N. Zhang H. (2007). Study of the inhibition effects of *Bifidobacterium* supernatants towards growth of *Bacillus cereus* and *Escherichia coli*. Int. J. Dairy Sci. 2, 116–125. 10.3923/ijds.2007.116.125

[B13] Chung W. S. Walker A. W. Louis P. Parkhill J. Vermeiren J. Bosscher D.. (2016). Modulation of the human gut microbiota by dietary fibres occurs at the species level. BMC Biol. 14:3. 10.1186/s12915-015-0224-3 26754945 PMC4709873

[B14] Clarke S. F. Murphy E. F. Nilaweera K. Ross P. R. Shanahan F. O'Toole P. W.. (2012). The gut microbiota and its relationship to diet and obesity: new insights. Gut Microbes 3, 186–202. 10.4161/gmic.20168 22572830 PMC3427212

[B15] Corbett A. McGowin A. Sieber S. Flannery T. Sibbitt B. (2012). A method for reliable voluntary oral administration of a fixed dosage (mg/kg) of chronic daily medication to rats. Lab. Anim. 46, 318–324. 10.1258/la.2012.012018 22969146

[B16] Cotter P. D. Stanton C. Ross R. P. Hill C. (2012). The impact of antibiotics on the gut microbiota as revealed by high throughput DNA sequencing. Discov. Med. 13, 193–199. 22463795

[B17] D'Alessio D. (2008). Intestinal hormones and regulation of satiety: the case for CCK, GLP-1, PYY, and Apo A-IV. J. Parenter. Enteral Nutr. 32, 567–568. 10.1177/0148607108322401 18753394

[B18] Davies E. A. Bevis H. E. Potter R. Harris J. Williams G. C. Delves-Broughton J. (1998). Research note: the effect of pH on the stability of nisin solution during autoclaving. Lett. Appl. Microbiol. 27, 186–187.

[B19] Delcour J. A. Bruneel C. Derde L. J. Gomand S. V. Pareyt B. Putseys J. A.. (2010). Fate of starch in food processing: from raw materials to final food products. Annu. Rev. Food Sci. Technol. 1, 87–111. 10.1146/annurev.food.102308.124211 22129331

[B20] Delves-Broughton J. (2005). Nisin as a food preservative. Food Aust. 57, 525–527. 10.4315/0362-028X-57.10.874 31121693

[B21] Denev S. A. (2006). Role of *Lactobacilli* in gastrointestinal ecosystem. Bulgarian J. Agric. Sci. 12, 63–114.

[B22] de Pablo M. A. Gaforio J. J. Gallego A. M. Ortega E. Galvez A. M. Alvarez de Cienfuegos López G. (1999). Evaluation of immunomodulatory effects of nisin-containing diets on mice. FEMS Immunol. Med. Microbiol. 24, 35–42. 10.1111/j.1574-695X.1999.tb01262.x 10340710

[B23] Desai K. G. (2005). Preparation and characteristics of high-amylose corn starch/pectin blend microparticles: a technical note. Aaps Pharmscitech. 6, E202–E208. 10.1208/pt060230 16353979 PMC2750533

[B24] De Vos P. Garrity G. M. Jones D. Krieg N. R. Ludwig W. Rainey F. A. (eds.). (2009). Bergey's Manual of Systematic Bacteriology. New York, NY: Springer.

[B25] Dimantov A. Greenberg M. Kesselman E. Shimoni E. (2004). Study of high amylose corn starch as food grade enteric coating in a microcapsule model system. Innov. Food Sci. Emerg. Technol. 5, 93–100. 10.1016/j.ifset.2003.11.003

[B26] Draper L. A. Cotter P. D. Hill C. Ross R. P. (2015). Lantibiotic resistance. Microbiol. Mol. Biol. Rev. 79, 171–191. 10.1128/MMBR.00051-14 25787977 PMC4394878

[B27] Edgar R. C. (2010). Search and clustering orders of magnitude faster than BLAST. Bioinformatics 26, 2460–2461. 10.1093/bioinformatics/btq461 20709691

[B28] Field D. Begley M. O'Connor P. M. Daly K. M. Hugenholtz F. Cotter P. D.. (2012). Bioengineered nisin A derivatives with enhanced activity against both gram positive and gram negative pathogens. PLOS ONE 7:e46884. 10.1371/journal.pone.0046884 23056510 PMC3466204

[B29] Forssell P. (2004). Starch-based microencapsulation, in Starch in Food, ed Eliasson A.-C. (Abington, UK: Woodhead Publishing), 461–473.

[B30] Freire C. Podczeck F. Ferreira D. Veiga F. Sousa J. Pena A. (2010). Assessment of the *in-vivo* drug release from pellets film-coated with a dispersion of high amylose starch and ethylcellulose for potential colon delivery. J. Pharm. Pharmacol. 62, 55–61. 10.1211/jpp.62.01.0005 20722999

[B31] Gharsallaoui A. Oulahal N. Joly C. Degraeve P. (2016). Nisin as a food preservative: part 1: physicochemical properties, antimicrobial activity, and main uses. Crit. Rev. Food Sci. Nutr. 56, 1262–1274. 10.1080/10408398.2013.763765 25675115

[B32] Gibson G. R. Wang X. (1994). Regulatory effects of bifidobacteria on the growth of other colonic bacteria. J. Appl. Bacteriol. 77, 412–420. 10.1111/j.1365-2672.1994.tb03443.x 7989269

[B33] Gómez-Gallego C. Pohl S. Salminen S. De Vos W. M. Kneifel W. (2016). *Akkermansia* muciniphila: a novel functional microbe with probiotic properties. Benef. Microbes 7, 571–584. 10.3920/BM2016.0009 27291403

[B34] Goodfellow M. Kämpfer P. Busse H.-J. Trujillo M. E. Suzuki K.-i. Ludwig W. (eds.). (2012). Bergey's Manual of Systematic Bacteriology. New York, NY: Springer.

[B35] Gough R. Gómez-Sala B. O'Connor P. M. Rea M. C. Miao S. Hill C.. (2017a). A simple method for the purification of nisin. Probiotics Antimicrob. Proteins 9, 363–369. 10.1007/s12602-017-9287-5 28555255

[B36] Gough R. O'Connor P. M. Rea M. C. Gómez-Sala B. Miao S. Hill C. (2017b). Simulated gastrointestinal digestion of nisin and interaction between nisin and bile. LWT Food Sci. Technol. 86, 530–537. 10.1016/j.lwt.2017.08.031

[B37] Gupta S. M. Aranha C. C. Reddy K. V. (2008). Evaluation of developmental toxicity of microbicide nisin in rats. Food Chem. Toxicol. 46, 598–603. 10.1016/j.fct.2007.09.006 17949878

[B38] Hagiwara A. Imai N. Nakashima H. Toda Y. Kawabe M. Furukawa F.. (2010). A 90-day oral toxicity study of nisin A, an anti-microbial peptide derived from *Lactococcus lactis* subsp lactis, in F344 rats. Food Chem. Toxicol. 48, 2421–2428. 10.1016/j.fct.2010.06.002 20621644

[B39] Haub M. D. Hubach K. L. Al-Tamimi E. K. Ornelas S. Seib P. A. (2010). Different types of resistant starch elicit different glucose reponses in humans. J. Nutr. Metab. 2010:230501. 10.1155/2010/230501 20700404 PMC2911581

[B40] Heinemann B. Williams R. (1966). Inactivation of nisin by pancreatin. J. Dairy Sci. 49, 312–314. 10.3168/jds.S0022-0302(66)87854-2 5960169

[B41] Hsu S. T. Breukink E. Tischenko E. Lutters M. A. G. de Kruijff B. Kaptein R.. (2004). The nisin-lipid II complex reveals a pyrophosphate cage that provides a blueprint for novel antibiotics. Nat. Struct. Mol. Biol. 11, 963–967. 10.1038/nsmb830 15361862

[B42] Jandhyala S. M. Talukdar R. Subramanyam C. Vuyyuru H. Sasikala M. Nageshwar Reddy D. (2015). Role of the normal gut microbiota. World J. Gastroenterol. 21, 8787–8803. 10.3748/wjg.v21.i29.8787 26269668 PMC4528021

[B43] Jarvis B. Mahoney R. R. (1969). Inactivation of nisin by alpha-chymotrypsin. J. Dairy Sci. 52, 1448–1450. 10.3168/jds.S0022-0302(69)86771-8 5369339

[B44] Javurek A. B. Spollen W. G. Johnson S. A. Bivens N. J. Bromert K. H. Givan S. A.. (2017). Consumption of a high-fat diet alters the seminal fluid and gut microbiomes in male mice. Reprod. Fertil. Dev. 29, 1602–1612. 10.1071/RD16119 27569192

[B45] Józefiak D. Kieronczyk B. Juśkiewicz J. Zdunczyk Z. Rawski M. Długosz J.. (2013). Dietary nisin modulates the gastrointestinal microbial ecology and enhances growth performance of the broiler chickens. PLoS ONE 8:e85347. 10.1371/journal.pone.0085347 24376878 PMC3869907

[B46] Kieronczyk B. Pruszynska-Oszmalek E. Swiatkiewicz S. Rawski M. Dlugosz J. Engberg R. M. (2016). The nisin improves broiler chicken growth performance and interacts with salinomycin in terms of gastrointestinal tract microbiota composition. J. Anim. Feed Sci. 25, 309–316. 10.22358/jafs/67802/2016

[B47] Kverka M. Zakostelska Z. Klimesova K. Sokol D. Hudcovic T. Hrncir T.. (2011). Oral administration of *Parabacteroides* distasonis antigens attenuates experimental murine colitis through modulation of immunity and microbiota composition. Clin. Exp. Immunol. 163, 250–259. 10.1111/j.1365-2249.2010.04286.x 21087444 PMC3043316

[B48] Lauková A. Chrastinová L. Plachá I. Kandricáková A. Szabóová R. Strompfová V.. (2014). Beneficial effect of lantibiotic nisin in rabbit husbandry. Probiotics Antimicrob. Proteins 6, 41–46. 10.1007/s12602-014-9156-4 24676766

[B49] Leahy S. C. Higgins D. G. Fitzgerald G. F. van Sinderen D. (2005). Getting better with Bifidobacteria. J. Appl. Microbiol. 98, 1303–1315. 10.1111/j.1365-2672.2005.02600.x 15916644

[B50] Le Blay G. Lacroix C. Zihler A. Fliss I. (2007). *In vitro* inhibition activity of nisin A, nisin Z, pediocin PA-1 and antibiotics against common intestinal bacteria. Lett. Appl. Microbiol. 45, 252–257. 10.1111/j.1472-765X.2007.02178.x 17718835

[B51] Le Lay C. Fernandez B. Hammami R. Ouellette M. Fliss I. (2015). On *Lactococcus lactis* UL719 competitivity and nisin (Nisaplin ®) capacity to inhibit *Clostridium* difficile in a model of human colon. Front. Microbiol. 6:1020. 10.3389/fmicb.2015.01020 26441942 PMC4585240

[B52] Lillie L. E. Temple N. J. Florence L. Z. (1996). Reference values for young normal Sprague-Dawley rats: weight gain, hematology and clinical chemistry. Hum. Exp. Toxicol. 15, 612–616. 10.1177/096032719601500802 8863053

[B53] Lockyer S. Nugent A. P. (2017). Health effects of resistant starch. Nutr. Bull. 42, 10–41. 10.1111/nbu.12244

[B54] Martínez I. Kim J. Duffy P. R. Schlegel V. L. Walter J. (2010). Resistant starches types 2 and 4 have differential effects on the composition of the fecal microbiota in human subjects. PLoS ONE 5:e15046. 10.1371/journal.pone.0015046 21151493 PMC2993935

[B55] McCleary B. V. McNally M. Rossiter P. (2002). Measurement of resistant starch by enzymatic digestion in starch and selected plant materials: collaborative study. J. AOAC Int. 85, 1103–1111. 12374410

[B56] Milojevic S. Newton J. M. Cummings J. H. Gibson G. R. Botham R. L. Ring S. G. (1996). Amylose as a coating for drug delivery to the colon: preparation and *in vitro* evaluation using 5-aminosalicylic acid pellets. J. Control. Release 38, 75–84. 10.1016/0168-3659(95)00112-3

[B57] Nicholson J. K. Holmes E. Kinross J. Burcelin R. Gibson G. Jia W.. (2012). Host-gut microbiota metabolic interactions. Science 336, 1262–1267. 10.1126/science.1223813 22674330

[B58] Noor S. O. Ridgway K. Scovell L. Kemsley E. K. Lund E. K. Jamieson C.. (2010). Ulcerative colitis and irritable bowel patients exhibit distinct abnormalities of the gut microbiota. BMC Gastroenterol. 10:134. 10.1186/1471-230X-10-134 21073731 PMC3002299

[B59] Oksanen J. Blanchet F. G. Kindt R. Legendre P. Minchin P. O'Hara R. B. (2015). Vegan: Community Ecology Package, Version 2.2-1. Available online at: https://CRAN.R-project.org/package=vegan

[B60] Ozdogan M. Ustundag A. O. (2015). Effects of bacteriocin and organic acids on growth performance of Japanese quails. Sci. Papers Series D Anim. Sci. 58, 164–169.

[B61] Pu H. Y. Chen L. Li X. Xie F. Yu L. Li L. (2011). An oral colon-targeting controlled release system based on resistant starch acetate: synthetization, characterization, and preparation of film-coating pellets. J. Agric. Food Chem. 59, 5738–5745. 10.1021/jf2005468 21513356

[B62] R Core Team (2015). R: A Language and Environment for Statistical Computing. Vienna: R Foundation for Statistical Computing Available online at: https://www.r-project.org/

[B63] Rea M. C. Alemayehu D. Casey P. G. O'Connor P. M. Lawlor P. G. Walsh M.. (2014). Bioavailability of the anti-clostridial bacteriocin thuricin CD in gastrointestinal tract. Microbiology 160, 439–445. 10.1099/mic.0.068767-0 24287693

[B64] Recife A. C. D. Meneguin A. B. Cury B. S. F. Evangelista R. C. (2017). Evaluation of retrograded starch as excipient for controlled release matrix tablets. J. Drug Deliv. Sci. Technol. 40, 83–94. 10.1016/j.jddst.2017.06.003

[B65] Reddy K. V. Gupta S. M. Aranha C. C. (2011). Effect of antimicrobial peptide, nisin, on the reproductive functions of rats. ISRN Vet. Sci. 2011:828736. 10.5402/2011/828736 23738116 PMC3658505

[B66] Reddy K. V. Aranha C. Gupta S. M. Yedery R. D. (2004). Evaluation of antimicrobial peptide nisin as a safe vaginal contraceptive agent in rabbits: *in vitro* and *in vivo* studies. Reproduction 128, 117–126. 10.1530/rep.1.00028 15232069

[B67] Reeves A. E. Koenigsknecht M. J. Bergin I. L. Young V. B. (2012). Suppression of *Clostridium* difficile in the gastrointestinal tracts of germfree mice inoculated with a murine isolate from the family *Lachnospiraceae*. Infect. Immun. 80, 3786–3794. 10.1128/IAI.00647-12 22890996 PMC3486043

[B68] Ryan M. P. Rea M. C. Hill C. Ross R. P. (1996). An application in cheddar cheese manufacture for a strain of *Lactococcus lactis* producing a novel broad-spectrum bacteriocin, lacticin 3147. Appl. Environ. Microbiol.. 62, 612–619. 8593062 10.1128/aem.62.2.612-619.1996 PMC167827

[B69] Sajilata M. G. Singhal R. S. Kulkarni P. R. (2006). Resistant starch - a review. Compr. Rev. Food Sci. Food Saf. 5, 1–17. 10.1111/j.1541-4337.2006.tb00076.x 33412740

[B70] Schmieder R. Edwards R. (2011). Quality control and preprocessing of metagenomic datasets. Bioinformatics 27, 863–864. 10.1093/bioinformatics/btr026 21278185 PMC3051327

[B71] Scott K. P. Antoine J. M. Midtvedt T. van Hemert S. (2015). Manipulating the gut microbiota to maintain health and treat disease. Microb. Ecol. Health Dis. 26:25877. 10.3402/mehd.v26.25877 25651995 PMC4315778

[B72] Schubert A. M. Sinani H. Schloss P. D. (2015). Antibiotic-induced alterations of the murine gut microbiota and subsequent effects on colonization resistance against Clostridium difficile. mBio 6:4. 10.1128/mBio.00974-15 26173701 PMC4502226

[B73] Shen J. Liu Z. Yu Z. Zhu W. (2017). Monensin and nisin affect rumen fermentation and microbiota differently *in vitro*. Front. Microbiol. 8:1111. 10.3389/fmicb.2017.01111 28670304 PMC5472720

[B74] Shtenberg A. J. Ignatev A. D. (1970). Toxicological evaluation of some combinations of food preservatives. Food Cosmet. Toxicol. 8, 369–380. 10.1016/S0015-6264(70)80390-X 5489399

[B75] Shu Q. Gill H. S. (2001). A dietary probiotic (*Bifidobacterium* lactis HN019) reduces the severity of *Escherichia coli* O157:H7 infection in mice. Med. Microbiol. Immunol. 189, 147–152. 10.1007/s430-001-8021-9 11388612

[B76] Sibley E. (2004). Carbohydrate digestion and absorption, in Encyclopedia of Gastroenterology, ed Johnson L. R. (New York, NY: Elsevier), 275–278.

[B77] Situ W. Chen L. Wang X. Li X. (2014). Resistant starch film-coated microparticles for an oral colon-specific polypeptide delivery system and its release behaviors. J. Agric. Food Chem. 62, 3599–3609. 10.1021/jf500472b 24684664

[B78] Slootweg J. C. Liskamp R. M. Rijkers D. T. (2013). Scalable purification of the lantibiotic nisin and isolation of chemical/enzymatic cleavage fragments suitable for semi-synthesis. J. Peptide Sci. 19, 692–699. 10.1002/psc.2551 24023046

[B79] Suzuki T. A. Nachman M. W. (2016). Spatial heterogeneity of gut microbial composition along the gastrointestinal tract in natural populations of house mice. PLoS ONE 11:e0163720. 10.1371/journal.pone.0163720 27669007 PMC5036816

[B80] Tachon S. Zhou J. N. Keenan M. Martin R. Marco M. L. (2013). The intestinal microbiota in aged mice is modulated by dietary resistant starch and correlated with improvements in host responses. FEMS Microbiol. Ecol. 83, 299–309. 10.1111/j.1574-6941.2012.01475.x 22909308

[B81] US Food and Drug Administration (1988). Nisin preparation; affirmation of GRAS status as a direct human food ingredient. Fed. Regist. 53, 11247–11251.

[B82] van Staden D. A. Brand A. M. Endo A. Dicks L. M. (2011). Nisin F, intraperitoneally injected, may have a stabilizing effect on the bacterial population in the gastro-intestinal tract, as determined in a preliminary study with mice as model. Lett. Appl. Microbiol. 53, 198–201. 10.1111/j.1472-765X.2011.03091.x 21609345

[B83] Vázquez-Baeza Y. Pirrung M. Gonzalez A. Knight R. (2013). EMPeror: a tool for visualizing high-throughput microbial community data. Gigascience 2:16. 10.1186/2047-217X-2-16 24280061 PMC4076506

[B84] Vital M. Howe A. C. Tiedje J. M. (2014). Revealing the bacterial butyrate synthesis pathways by analyzing (meta)genomic data. MBio 5:e00889–14. 10.1128/mBio.00889-14 24757212 PMC3994512

[B85] Walter J. (2008). Ecological role of *Lactobacilli* in the gastrointestinal tract: implications for fundamental and biomedical research. Appl. Environ. Microbiol. 74, 4985–4996. 10.1128/AEM.00753-08 18539818 PMC2519286

[B86] Wang Q. Garrity G. M. Tiedje J. M. Cole J. R. (2007). Naive Bayesian classifier for rapid assignment of rRNA sequences into the new bacterial taxonomy. Appl. Environ. Microbiol. 73, 5261–5267. 10.1128/AEM.00062-07 17586664 PMC1950982

[B87] Wang S. J. Li C. L. Copeland L. Niu Q. Wang S. (2015). Starch retrogradation: a comprehensive review. Compr. Rev. Food Sci. Food Saf. 14, 568–585. 10.1111/1541-4337.12143

[B88] Wiessing K. R. Xin L. Budgett S. C. Poppitt S. D. (2015). No evidence of enhanced satiety following whey protein- or sucrose-enriched water beverages: a dose response trial in overweight women. Eur. J. Clin. Nutr. 69, 1238–1243. 10.1038/ejcn.2015.107 26130302

[B89] Willing B. P. Russell S. L. Finlay B. B. (2011). Shifting the balance: antibiotic effects on host-microbiota mutualism. Nat. Rev. Microbiol. 9, 233–243. 10.1038/nrmicro2536 21358670

[B90] Wilson P. J. Basit A. W. (2005). Exploiting gastrointestinal bacteria to target drugs to the colon: an *in vitro* study using amylose coated tablets. Int. J. Pharm. 300, 89–94. 10.1016/j.ijpharm.2005.05.010 16023805

[B91] Younes M. Aggett P. Aguilar F. Crebelli R. Dusemund B. Filipič M. (2017). Safety of nisin (E 234) as a food additive in the light of new toxicological data and the proposed extension of use. EFSA J. 15:5063 10.2903/j.efsa.2017.5063 PMC7009836 32625365

[B92] Yu Y. South T. Huang X. F. (2009). Inter-meal interval is increased in mice fed a high whey, as opposed to soy and gluten, protein diets. Appetite 52, 372–379. 10.1016/j.appet.2008.11.011 19100798

[B93] Zeng B. Han S. Wang P. Wen B. Jian W. Guo W.. (2015). The bacterial communities associated with fecal types and body weight of rex rabbits. Sci. Rep. 5:9342. 10.1038/srep09342 25791609 PMC4366860

[B94] Zhou H. Fang J. Tian Y. Lu X. Y. (2014). Mechanisms of nisin resistance in Gram-positive bacteria. Ann. Microbiol. 64, 413–420. 10.1007/s13213-013-0679-9

[B95] Zhou J. Martin R. J. Tulley R. T. Raggio A. M. McCutcheon K. L. Shen L.. (2008). Dietary resistant starch upregulates total GLP-1 and PYY in a sustained day-long manner through fermentation in rodents. Am. J. Physiol. Endocrinol. Metabol. 295, E1160–E1166. 10.1152/ajpendo.90637.2008 18796545 PMC2584810

